# Supplementary material for: Complexome profiling on the Chlamydomonas lpa2 mutant reveals insights into PSII biogenesis and new PSII associated proteins
Source: J Exp Bot. 2021 Aug 26;73(1):245–62. doi: 10.1093/jxb/erab390 (PMC8730698; doi:10.1093/jxb/erab390)
Supplement: erab390_suppl_Supplementary_Dataset_S1 [file erab390_suppl_supplementary_dataset_s1.zip › Supplemental Dataset 1 - Excel List and all profiles/plots/VPS45_Cre07.g333950.html]

Trivial name: VPS45  
  
Euclidean distance: 4049.30  
Mean Intensity (WT): 0.00  
Mean Intensity (Mut): 124.95  
Distance: 32.41  
  
MapMan: protein.targeting.secretory pathway.vacuole  
  
p value of intensity sums Welch test: 0.4226
